# Club cell protein 16 in sera from trauma patients modulates neutrophil migration and functionality via CXCR1 and CXCR2

**DOI:** 10.1186/s10020-019-0115-0

**Published:** 2019-10-30

**Authors:** Baolin Xu, Andrea Janicova, Jan Tilmann Vollrath, Philipp Störmann, Lukas Martin, Ingo Marzi, Sebastian Wutzler, Frank Hildebrand, Sabrina Ehnert, Borna Relja

**Affiliations:** 1 Department of Trauma, Hand and Reconstructive Surgery, University Hospital Frankfurt, Goethe University, Frankfurt, Germany; 2 Department of Radiology and Nuclear Medicine, Experimental Radiology, Otto von Guericke University Magdeburg, Magdeburg, Germany; 3 Department of Intensive and Intermediate Care, Medical Faculty, RWTH Aachen University, Aachen, Germany; 4 Department of Trauma, Hand and Orthopedic Surgery, Helios Horst Schmidt Clinic, Wiesbaden, Germany; 5 Department of Orthopaedic Trauma, University Clinic RWTH Aachen, Aachen, Germany; 6 Department of Trauma and Reconstructive Surgery, Siegfried Weller Research Institute, Eberhard Karls University Tuebingen, BG Trauma Center Tuebingen, Tuebingen, Germany

**Keywords:** Trauma, Pneumonia, Neutrophils, Migration, ROS, Phagocytosis, Apoptosis

## Abstract

**Background:**

Club Cell protein (CC)16 correlates with lung injury and respiratory complications, which are in part triggered by polymorphonuclear leukocytes (PMNL) in severely traumatized patients (TP). CC16 exerts anti-inflammatory and immunosuppressive effects, however, its influence on PMNL functions after trauma is unknown. Here, we evaluated whether CC16 present in sera from TP could modify the biological functions of PMNL.

**Methods:**

Sera from 16 severely injured TP without pneumonia (no P, *n* = 8) or with pneumonia (P, n = 8) were collected at admission to emergency department (ED) and 1 day prior pneumonia and pre-incubated with or without anti-CC16 antibody for CC16 neutralization. Samples from the equal post-injury days in the corresponding no P group were used. Neutrophils were isolated from healthy volunteers (HV, *n* = 5) and incubated with 20% of the serum medium from TP, respectively. In PMNL, CD62L, CD11b/CD18 and CD31 expression, migratory capacity, phagocytosis rate, oxidative burst and apoptosis were investigated. In isolated PMNL, CXCR1 and CXCR2 were neutralized before stimulation with CC16, and oxidative burst, phagocytosis and apoptosis were analyzed in neutrophils and their subsets.

**Results:**

Serum from the P group enhanced significantly PMNL migration compared to no P group, while CC16-neutralization further increased the migratory rate of PMNL in both groups. CC16-neutralization increased significantly the expression of CD62L in the P group at ED. Oxidative burst was significantly increased in the P group vs. no P during the study period. CC16 seemed to have no influence on oxidative burst and phagocytosis in TP. However, in a more controlled study design, CC16 induced a significant increase of oxidative burst and a decrease of apoptosis of CD16^+^ granulocytes. These effects were markedly observed in mature CD16^bright^CD62L^bright^ and immune suppressive CD16^bright^CD62L^dim^ neutrophils. In mature subset, CXCR1 and CXCR2 neutralization diminished CC16-induced effects.

**Conclusions:**

CC16 in sera from multiply traumatized patients, notably of those with pneumonia, has significant effects on PMNL. The results suggest an association of CC16 with CXCR1 and CXCR2. Our data suggest that CC16 reduces the migratory capacity of PMNL and thus modulates their function in patients with respiratory complications after trauma.

## Introduction

Trauma is one of the leading causes of mortality and an important contributing factor to disability worldwide (Sadeghi-Bazargani et al. 2018; Martines et al. 2018). Adjacent to the main causes of immediate or early post-injury death e.g. massive bleeding or severe brain injuries, infectious complications such as pneumonia, sepsis or multiple organ failure (MOF) triggered by inflammatory changes provoke late mortality during the hospital stay (Liodaki et al. 2015).

Traumatic injury initiates the release of endogenous damage-associated molecular patterns such as high mobility group box 1 or interleukin (IL)-1 (Eigenbrod et al. 2008; Scaffidi et al. 2002), as well as pathogen-associated molecular pattern such as lipopolysaccharide or heptose-1,7-bisphosphate (Wang et al. 2018; Gaudet et al. 2015) causing a proinflammatory response. This reaction is closely associated with an excessive release of further cytokines such as IL-8, tumor necrosis factor (TNF)-α, IL-6, and IL-1β (Hauser and Otterbein 2018; Zhang et al. 2010a; Zhang et al. 2010b), and with the activation and recruitment of polymorphonuclear leukocytes (PMNL) with their subsequently increased immigration into tissues (Relja et al. 2013; Wagner et al. 2018). PMNL, constituting innate effector cells of the first line of defense after trauma, mediate the anti-microbial and post-injury immune response, and thereby play an important role in the progress of inflammation, contributing to the development of pneumonia (Rosales et al. 2016; Nam et al. 2018). With regard to their specific functions after trauma, a decrease of endothelial adhesion molecule L-selectin (CD62L), which initiates rolling and capturing process, and modulations of other adhesion molecules, were associated with pulmonary complications after trauma (Relja et al. 2016; Sakamaki et al. 1995; Donnelly et al. 1994; Bahra et al. 1998). Furthermore, enhanced migration of neutrophils infiltrating the lung tissue has been linked to increased pulmonary IL-8 concentration in trauma patients suffering from pneumonia or acute respiratory distress syndrome (ARDS) (Relja et al. 2016; Pallister et al. 2002; Bhatia et al. 2005). Adjacent to the changes in adhesion molecules and migratory behavior, after severe trauma, phagocytosis and oxidative burst activity of granulocytes is modulated (Sturm et al. 2017; Kanyilmaz et al. 2013; Liao et al. 2013). While several studies have shown that the migratory ability of neutrophils and production of reactive oxygen species (ROS) are strongly increased after trauma (Relja et al. 2016; Winterbourn et al. 2016; Mittal et al. 2014), a delayed apoptosis upon incubation with sera from septic patients was observed (Shen et al. 2017; Guo et al. 2006). Under the condition of acute inflammation, three neutrophil subsets can be found in the blood: cells with a conventional segmented nucleus, with a banded nucleus, and T-cell-suppressing CD62L^dim^ with a high number of nuclear lobes, termed mature, banded and hypersegmented neutrophils, respectively (Silvestre-Roig et al. 2019; Pillay et al. 2012). As neutrophils age, they change their phenotype but also their functions (Nagase et al. 2002; Martin et al. 2003; Rankin 2010). Thus, although neutrophils have been generally considered as a relatively homogeneous population for several decades, evidence for their true heterogeneity is emerging.

Club cell protein (CC)16 is a 15.8 kDa protein secreted by club cells along the tracheobronchial tree and especially in the distal respiratory bronchioles (Komaromy and Tigyi 1988; Bernard and Lauwerys 1995). This protein increasingly appears to protect the respiratory tract against oxidative stress and inflammation, since most studies have found associations between CC16 levels and the severity of major lung insults. Lung injury correlates with increased systemic CC16 levels in severely injured trauma patients (Wutzler et al. 2011). Moreover, its secondary increase indicated the development of pneumonia after trauma (Wutzler et al. 2012; Negrin et al. 2017). Next to the clinical biomarker characteristic to predict pneumonia after trauma, there is accumulating evidence that CC16 exerts pathophysiologically relevant effects for the development of pulmonary complications, since it may reduce the production of proinflammatory cytokines (Pang et al. 2017; Pang et al. 2015). Thus, CC16 exerts anti-inflammatory functions, which have been protective also in the development of other pulmonary diseases (Laucho-Contreras et al. 2015).

Since PMNL play a center role in post-traumatic inflammation, the influence of CC16 on their biological and physiological functions during the development of pneumonia may be relevant for understanding the disease pathophysiology and improving trauma care. Therefore, we evaluated how CC16, which is present in sera from traumatized patients with or without pneumonia will modify the functions of PMNL.

## Material and methods

### Patients

In this experimental trial, 16 severely injured trauma patients with a history of acute blunt or penetrating trauma and an injury severity score (ISS) ≥ 16 and five healthy volunteers were enrolled. Individuals that were younger 18 or older 80 years of age, suffering from severe burn injury, acute myocardial infarction, cancer or chemotherapy, HIV, infectious hepatitis, acute CMV infection and/or thromboembolic events, or receiving immunosuppressive drug therapy were excluded. The ISS was calculated according to the abbreviated injury scale (AIS) upon arrival at the emergency department (ED) (Palmer et al. 2013). The patients were included consecutively according to the inclusion and exclusion criteria for 24 months. Then, out of 167 patients, 16 matched pairs were created. After frequency-matching of patients according to the ISS and age, patients were grouped to the cohort without pneumonia (no P, *n* = 8) or with pneumonia (*P*, *n* = 8). The presence of pneumonia was defined by radiologic, clinical and bacteriologic findings with the presence of new pulmonary infiltrates on chest X-ray and at least one of the following criteria: positive blood culture, bronchial alveolar lavage and/or sputum culture.

### Blood sampling

Immediately after admission to the ED and daily for 10 days after trauma, blood samples were withdrawn in S-Manovette® Z-Gel tubes (Sarstedt, Nürmbrecht, Germany). After centrifugation at 2000 x g for 15 min at 4 °C the supernatants were stored at − 80 °C for further in vitro experiments with isolated neutrophils from healthy volunteers (HV). The samples that were used are those obtained at the admission to the ED and 1 day prior diagnosis of pneumonia (− 1 day prior pneumonia) or at the same day in the corresponding group of patients without pneumonia (no P) (− 1 day prior pneumonia).

Blood samples from HV were withdrawn in heparinized tubes (Sarstedt, Nürmbrecht, Germany) and kept at room temperature until isolation of PMNL.

### Isolation of PMNL

To isolate blood neutrophils the density-gradient centrifugation (Polymorphprep, density: 1.113 ± 0.001 g/ml, Axis-Shield, Oslo, Norway) was applied according to manufacturer’s instructions. Briefly, 4 ml of Polymorphprep were covered carefully with 4 ml heparinized blood from HV and centrifuged for 30 min. Then, the PMNL cell fraction was transferred to another tube for washing procedure with phosphate buffered saline w/o Ca^2+^ and Mg^2+^ (PBS, Invitrogen). The number of PMNL as well as their viability were determined by the trypan blue exclusion assay. The cells were cultured in RPMI 1640 medium (Seromed, Berlin, Germany; polypropylene tube, BD Bioscience, Franklin Lakes, NJ, USA) supplemented with 10% heat-inactivated fetal calf serum (FCS), 100 IU/mL penicillin, and 100 μg/mL streptomycin (Gibco, Karlsruhe, Germany) and 20 mM HEPES buffer (Sigma, Deisenhofen, Germany). Only cell cultures with a purity of > 95% were utilized for experimental use. For the analysis of the effects of sera obtained from TP, PMNL were cultured in media containing 20% of TP serum as described below.

### Cell stimulation

In vitro experiments with isolated neutrophils from healthy volunteers (HV) included the incubation of cells either with IL-8 (10 ng/ml, R&D Systems, Wiesbaden), the recombinant human CC16 (CC16_I: 100 ng/ml, CC16_II: 33.33 ng/ml and CC16_III: 1 ng/ml, respectively, R&D Systems) or with sera from TP that were obtained at the admission to the ED or 1 day prior to diagnosis of pneumonia (− 1 day prior pneumonia) or at the same day in the corresponding group of patients without pneumonia (no P) (− 1 day prior pneumonia). The serum: media ratio was 1: 5. Furthermore, sera from TP were either pre-incubated with or without anti-CC16-antibody for CC16-neutralization (10 ng/ml, human uteroglobin affinity purified Ab, R&D Systems). Additional in vitro analyses included the neutralization of CXCR1 or CXCR2, respectively, with antibodies (1 μg/ml, Human CXCR1/IL-8 RA Antibody and Human CXCR2/IL-8 RB Antibody; R&D Systems) or with the corresponding isotype controls (1 μg/ml, Mouse IgG2A Isotype Control; R&D Systems) for 1 hour prior stimulation of the cells with CC16 (100 ng/ml) for another hour.

### Migration assay

Migration capacity of isolated PMNL was examined using modified Transwell 24-well chambers (Corning, New York, USA) with 3-μm pores. Before the evaluation of the migratory rate of neutrophils, cell number was equalized to 1 × 10^6^ vital cells/ml. Subsequently, 100 μl of the cell suspension were placed in the upper chamber. The lower chamber contained 500 μl RPMI 1640 with supplements and either IL-8 (10 ng/ml, R&D Systems), the recombinant human CC16 (CC16_I: 100 ng/ml, CC16_II: 33.33 ng/ml and CC16_III: 1 ng/ml, respectively, R&D Systems) or 20% serum obtained from TP. The sera from TP were either pre-incubated with or without anti-CC16-antibody for CC16-neutralization (10 ng/ml, human uteroglobin affinity purified Ab, R&D Systems). After incubation for 3 h at 37 °C and 5% CO_2_, the upper transwell chamber was removed and cells migrating to the lower chamber were collected and counted after their staining with Türks-solution (1: 1, Merck). The number of cells migrating into the lower compartment was quantified. The migratory capacity of control (ctrl) cells that were incubated without serum in the lower compartment was set as 100%.

### Measurement of cell surface receptor expression by flow cytometry

Isolated PMNL (2 × 10^6^ cells/ml) were transferred in 250 μl RPMI 1640 with supplements as described above including 20% serum from TP. Control samples were incubated in medium containing supplements without sera, and as an additional control IL-8 was applied to the medium. The samples were incubated at 37 °C and 5% CO_2_ for 2 hours. Subsequently, the samples were centrifuged at 2100 x g for 15 min, the supernatant was removed and the cells were transferred into polystyrene FACS tubes. Then, the samples were incubated with mouse anti-human CD62L APC (clone: DREG-56), mouse anti-human CD31 (clone: WM59) and mouse anti-human CD11b/Mac-1 PE (clone: ICRF44, all BD Pharmingen) antibodies for 30 min. After subsequent washing procedure with 4 ml PBS supplemented with 0.5% bovine serum albumin (FACS buffer) at 400 x g for 5 min, the supernatants were removed and the cells were resuspended in 200 μl FACS buffer. Flow cytometric analyses of cell surface markers on a minimum of 20.000 cells was performed using BD FACS Canto 2*™* and FACD DIVA™ software (BD). The neutrophils were gated by the corresponding forward and side scatter scan (FSC and SSC) as shown in Fig. 2 a. Mean fluorescence units (MFU) were detected.

### Oxidative burst

Isolated PMNL (2 × 10^6^ cells/ml) were cultured as described above with or without 20% sera and with or without CC16-neutralization, respectively, or with IL-8. Control samples were incubated in culture medium. The samples were incubated at 37 °C and 5% CO_2_ for 2 h and subsequently centrifuged at 2100 x g for 15 min. Cell pellet was resuspended in culture medium (1 ml) with supplements and 100 μl of the cell suspension were transferred into polystyrene FACS tubes.

In a second in vitro experiment, isolated neutrophils were incubated with anti-CXCR1 or CXCR-2 antibodies for the neutralization of CXCR1 or CXCR2, respectively, or with the corresponding isotype controls (1 μg/ml) for 1 hour prior to the stimulation of the cells with CC16 (100 ng/ml) for another hour. Then, the samples were washed with FACS buffer and incubated with mouse anti-human CD62L PE-Cy7 (clone: DREG-56, BioLegend) and mouse anti-human CD16 AF647 (clone: 3G8, BD Pharmingen) antibodies for 30 min. After subsequent washing procedure with FACS buffer as described above, the supernatants were removed and cells were resuspended in 100 μl FACS buffer.

In both in vitro experiments, 20 μl CM-H_2_DCFDA (CM-H_2_DCFDA, General Oxidative Stress Indicator Kit, Invitrogen, Darmstadt, Germany) were added to each sample and the samples were incubated for 30 min at 37 °C and 5% CO_2_. Thereafter, 400 μl of the culture medium were added to each sample. After 1 hour at 37 °C and 5% CO_2_, cells were washed with FACS buffer, the supernatants were removed, and cells were resuspended in 200 μl FACS buffer for subsequent flow cytometric analyses as described above. The gating was performed as shown in Fig. 3 a. According to manufacturer’s protocol, the negative populations were set according to the CD16^+^ ROS-negative population. Then, the percentage of positive cells for oxidative stress and the MFU were determined.

### Phagocytosis

Isolated neutrophils were cultured as described above. Instead of applying the CM-H_2_DCFDA, here, pHrodo™ Red *Staphylococcus aureus* Bioparticles® Conjugate for Phagocytosis (Invitrogen, Darmstadt) were used. 100 μl pHrodo™ Red *Staphylococcus aureus* Bioparticles® were added to each sample and the samples were incubated for 1 hour at 37 °C and 5% CO_2_. Thereafter, cells were washed with FACS buffer, the supernatants were removed, and cells were resuspended in 200 μl FACS buffer for subsequent flow cytometric analyses as described above. The gating was performed as indicated for ROS production in Fig. 3 a with the difference that phagocytosis negative CD16^+^ cells were applied for the settings. The percentage of positive cells for phagocytosis and the MFU were determined.

### Apoptosis

Isolated neutrophils were cultured as described above. Instead of applying the CM-H_2_DCFDA, here, *Annexin V: FITC Apoptosis Detection Kit I* (BD Pharmingen) was used. 5 μl Annexin V-FITC and 5 μl Propidiumiodid were added to 100 μl of each sample and the samples were incubated for 15 min at room temperature. Thereafter, cells were washed with FACS buffer, the supernatants were removed, and cells were resuspended in 200 μl FACS buffer for subsequent flow cytometric analyses as described above. The gating was performed as indicated for ROS production in Fig. 3 a with the difference that apoptosis negative CD16^+^ cells were applied for the settings. The percentage of apoptotic cells and the MFU were determined.

### Statistical analysis

The statistical analyses were performed by using GraphPad Prism 6.0 software (GraphPad Software Inc. San Diego, CA). Data are given as mean ± standard error of the mean (SEM). Kruskal-Wallis with a Dunn post-hoc test was used for comparison among different groups. A *p* value below 0.05 was considered statistically significant.

## Results

### CC16 reduces the migration of isolated neutrophils towards pro-inflammatory chemoattractants

**Fig. 1:**
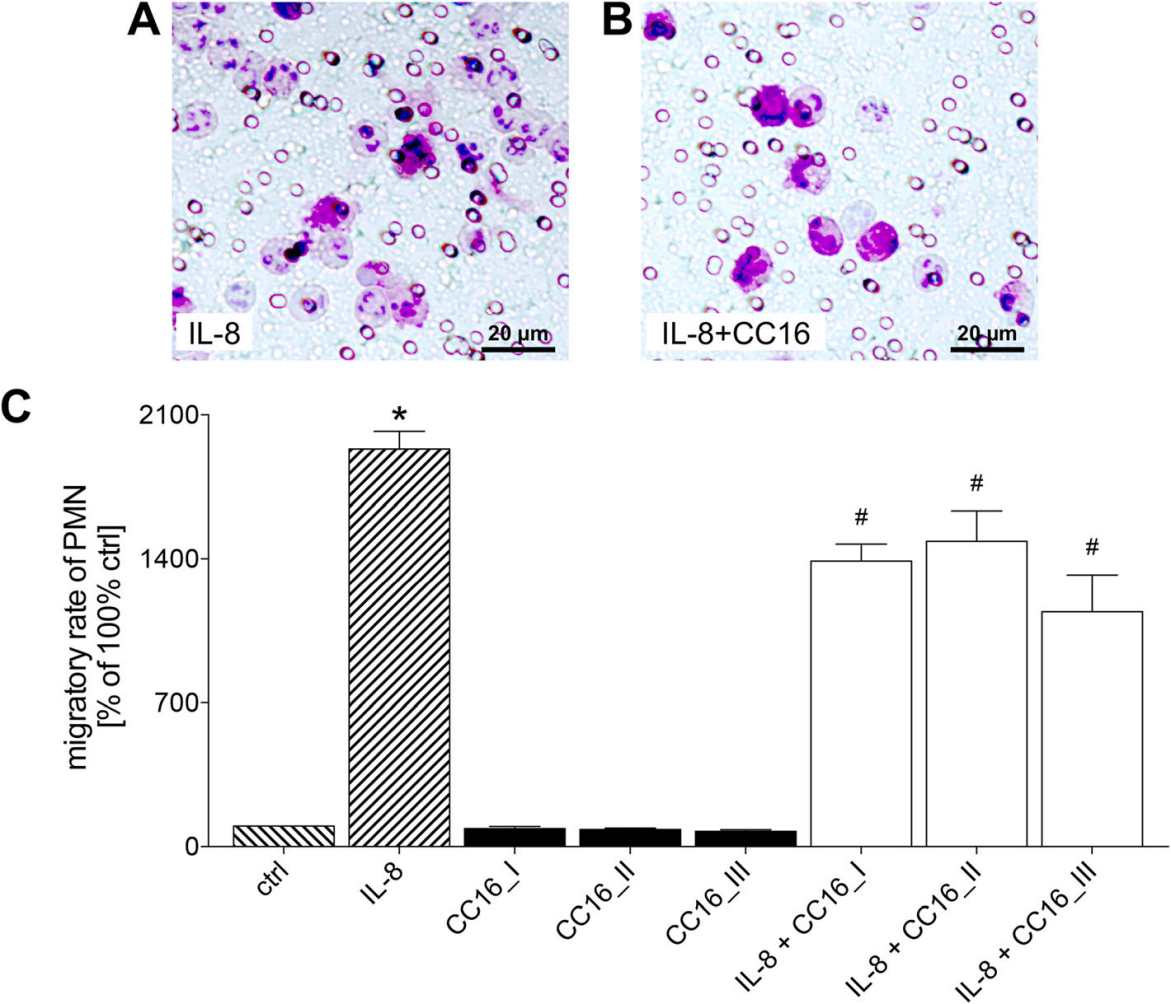
CC16 reduces PMNL migration towards IL-8. Isolated PMNL that have migrated to the lower side of the membrane. **a** IL-8 and (**b**) IL-8 concurrent with CC16 as chemoattractants.(**c**) Migration capacity of isolated PMN towards different concentrations of CC16 (CC16_I: 100 ng/ml, CC16_II: 33.33 ng/ml and CC16_III: 1 ng/ml, respectively). *p* < 0.05: *: vs. all; #: vs. ctrl, IL-8 and CC16I, II, and III

IL-8 administration in the lower transwell chamber induces a significant increase in PMNL migration from the upper compartment towards IL-8 compared to controls (*p* < 0.05, Fig. 1). Addition of CC16 alone to the medium in the lower compartment did not markedly change migration rates compared to control. No effects regarding the dose of CC16 were observed either. However, the concurrent application of IL-8 and CC16 in the lower compartment significantly decreased migration rates of PMNL compared with untreated controls and with IL-8 induced migration rates (*p* < 0.05, Fig. 1).

**Fig. 2:**
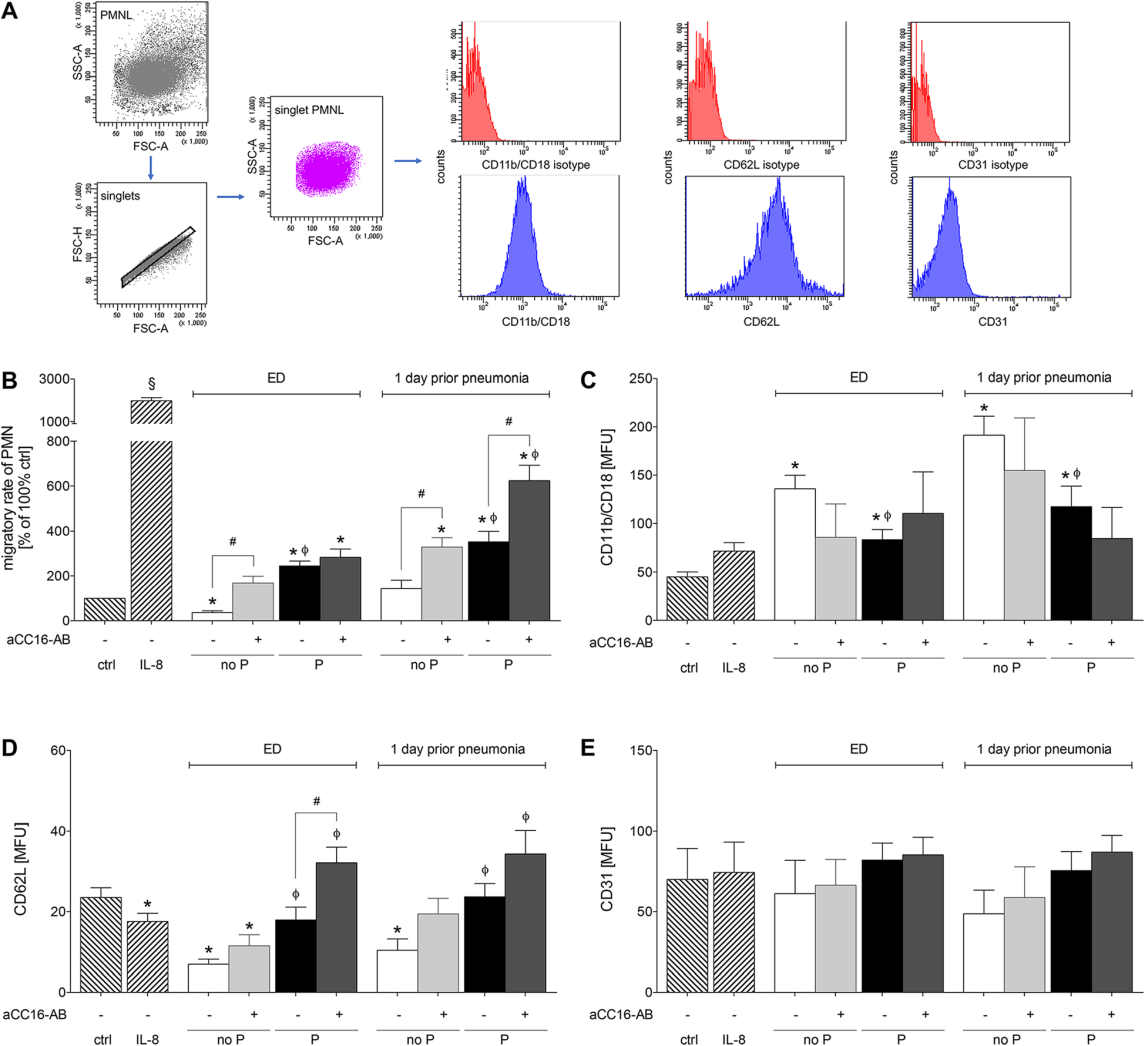
Increased PMNL migration upon CC16 neutralization is associated with concurrently enhanced CD62L. **a** Gating strategy of isolated PMNL and representative stainings for isotype or anti CD11b/CD18, CD62L and CD31 antibodies for the MFU evaluation is shown. Migratory rate of PMNL and CD11b/CD18, CD31, CD62L expression on PMNL after their incubation with serum samples from severely injured trauma patients. Patients were grouped to no P group without pneumonia or P group with pneumonia. Samples were obtained at admission to emergency department (ED) and one (Sadeghi-Bazargani et al. 2018) day prior to pneumonia. Samples from the equal post-injury days in the corresponding no P group were used. **b** Migratory rates of PMNL isolated from healthy volunteers towards IL-8 or sera from trauma patients with or without CC16 neutralization (aCC16-AB) is shown. **c** CD11b/CD18 expression, (**d**) CD62L expression and (**e**) CD31 expression on neutrophils is shown. Data are represented as mean ± SEM. *p* < 0.05: §: vs. all; *: vs. ctrl; Ф: P vs. corresponding no P group; #: CC16 vs. indicated

Migration of PMNL in the no P group was significantly lower vs. control, while the PMNL migration rate in the P group increased significantly vs. ctrl at ED (*p* < 0.05, Fig. 2 b). No significant changes in the PMNL migration rate in the no P group vs. ctrl were observed at 1 day prior pneumonia, while the PMNL migration was significantly increased in the P group vs. ctrl (*p* < 0.05, Fig. 2 b). Administration of sera from the P group to PMNL resulted in significantly higher migration rates of PMNL at ED as well as at 1 day prior pneumonia compared to the corresponding no P group (*p* < 0.05, Fig. 2 b). Notably neutralization of CC16 in both groups at 1 day prior pneumonia has shown a significantly increased PMNL migration capacity compared to the corresponding samples (*p* < 0.05 Fig. 2 b).

### CC16-reduced migratory rate of isolated neutrophils is associated with concurrently reduced CD62L

PMNL incubation with sera from TP without or with pneumonia significantly increased the surface expression of CD11b/CD18 vs. ctrl at ED as well as 1 day prior to pneumonia (*p* < 0.05, Fig. 2 c). Incubating isolated PMNL with sera from TP who developed pneumonia showed significantly decreased CD11b/CD18 expression at ED and at 1 day prior to pneumonia vs. corresponding no P group (*p* < 0.05, Fig. 2 c). Neutralization of CC16 has shown no significant changes regarding the surface expression of CD11b/CD18 on isolated PMNL (Fig. 2 c).

Surface expression of the CD62L on isolated PMNL in the no P group was significantly decreased compared to ctrl at ED and 1 day prior to pneumonia (*p* < 0.05, Fig. 2 d). CD62L expression in the P group was significantly increased vs. the corresponding no P group (*p* < 0.05, Fig. 2 d). However, there was no significant difference between the P group vs. ctrl. Neutralization of CC16 showed a trend to an increase in each group, however, CD62L expression was significantly increased only after CC16 neutralization in the P group vs. the corresponding sample at ED (*p* < 0.05, Fig. 2 d).

The surface expression of CD31 on isolated PMNL did not show any significant differences among the groups (Fig. 2 e).

### Blockade of chemokine receptors significantly reduces the CC16-induced oxidative stress in mature neutrophils

**Fig. 3:**
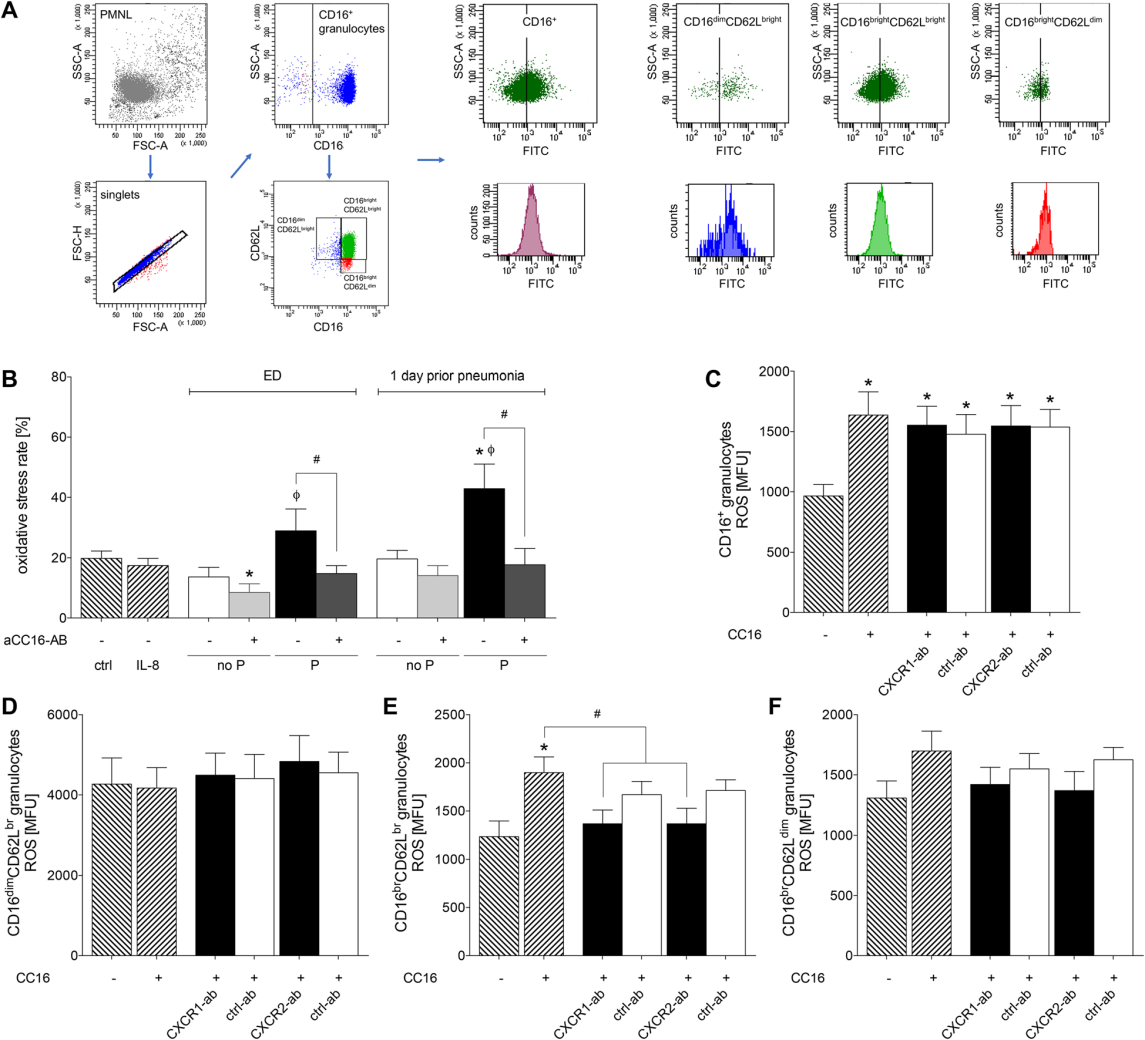
CC16-induced oxidative burst notably in the group of traumatized patients who develop complications is associated with CXCR1 and CXCR2 in mature neutrophils. **a** Gating strategy of isolated PMNL and representative stainings for CD16 and subsequent gating of CD16CD62L subsets with the exemplary MFU evaluation is shown. **b** Oxidative stress rate of PMNL after their incubation with IL-8 or sera samples from severely injured trauma patients. Patients were grouped to no P group without pneumonia or P group with to pneumonia. Samples were obtained at admission to emergency department (ED) and one (Sadeghi-Bazargani et al. 2018) day prior pneumonia. Samples from the equal post-injury days in the corresponding no P group were used. **c** Oxidative burst activity in CD16^+^ granulocytes after incubation with CC16 and neutralization of CXCR1 (CXCR1-ab), CXCR2 (CXCR2-ab) or the corresponding isotype control antibodies (ctrl-ab), respectively. **d** Oxidative burst activity of immature CD16^dim^CD62L^bright^, (**e**) mature CD16^bright^CD62L^bright^ and (**f**) immunesuppressive CD16^bright^CD62L^dim^ granulocytes. Data are represented as mean ± SEM. *p* < 0.05: *: vs. ctrl; #: CC16 vs. indicated

Incubation of isolated PMNL with IL-8 did not markedly change the percentage of the oxidative burst rate vs. untreated controls (Fig. 3 b). Oxidative burst rate after incubating PMNL with samples from the P group was significantly increased vs. ctrl at 1 day prior to pneumonia (*p* < 0.05, Fig. 3 b). Administration of sera from the P group to PMNL has resulted in significantly increased oxidative burst rate vs. the corresponding no P group at ED as well as at 1 day prior to pneumonia (*p* < 0.05, Fig. 3 b). Neutralization of CC16 in the no P group has shown a significant decrease in the no P group vs. ctrl at ED. A significant decrease in the oxidative burst rate has been observed in the P group at ED and at 1 day prior to pneumonia vs. corresponding sample as well (*p* < 0.05, Fig. 3 b).

With regard to the intensity of oxidative burst, a significant induction in CC16 stimulated granulocytes vs. ctrl has been observed (*p* < 0.05, Fig. 3 c). This increase in CC16 induced oxidative burst activity persisted among granulocytes upon CXCR1 as well as after CXCR2 neutralization (*p* < 0.05, Fig. 3 c). Further evaluation of neutrophil subsets with regard to oxidative burst has shown that there were no significant changes neither in immature CD16^dim^CD62L^bright^ nor in immune suppressive CD16^bright^CD62L^dim^ neutrophils (Fig. 3 e and f). However, oxidative burst activity has been significantly induced by CC16 in mature CD16^bright^CD62L^bright^ neutrophils vs. ctrl (*p* < 0.05, Fig. 3 e). In this subset, CXCR1 and CXCR2 neutralization resulted in significantly diminished oxidative burst as compared with CC16 stimulated samples (*p* < 0.05, Fig. 3 e).

### CC16 has no significant effects on phagocytosis behavior of isolated neutrophils

**Fig. 4:**
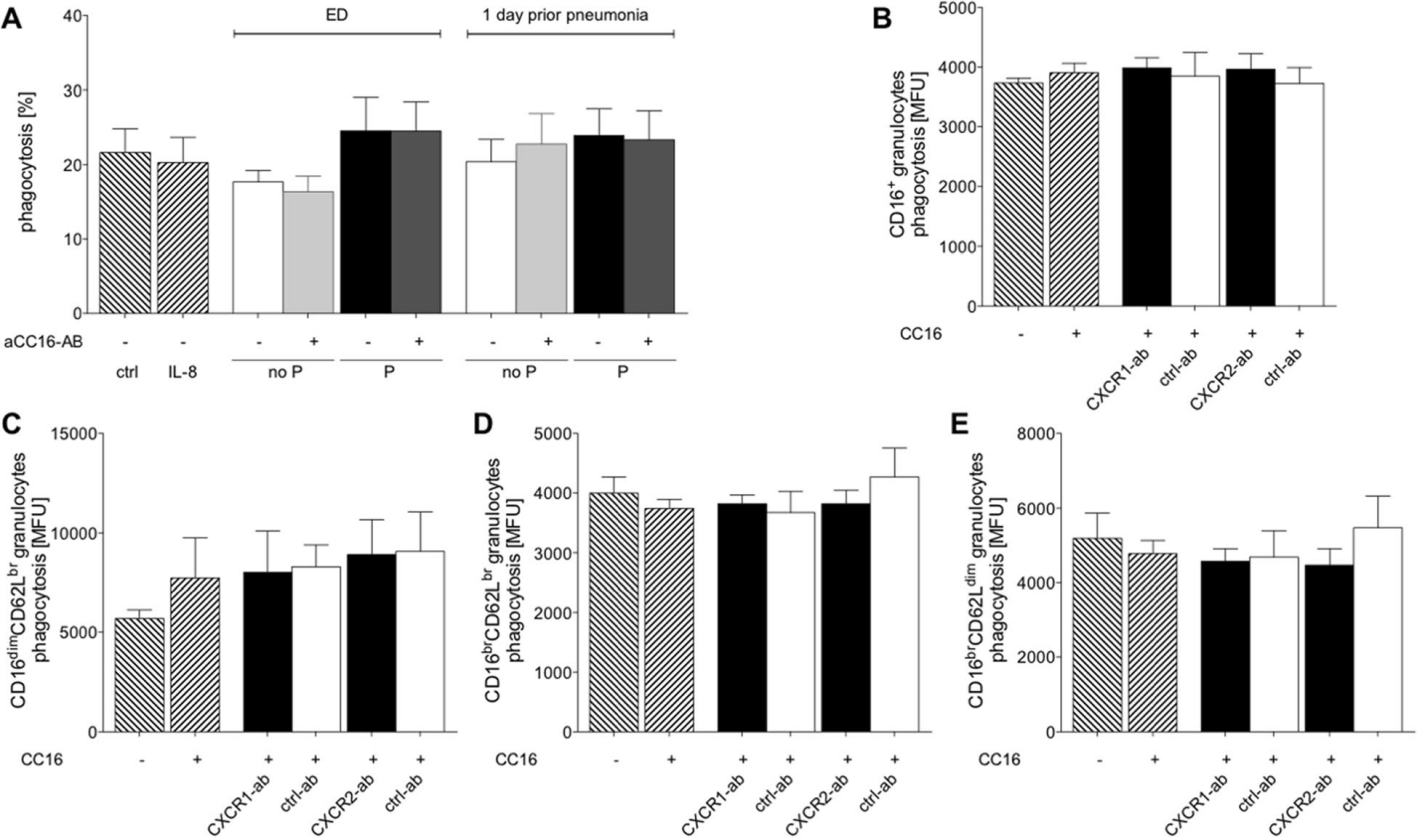
CC16 does not markedly influence phagocytosis in isolated PMNL. **a** Phagocytosis of PMNL after their incubation with IL-8 or sera samples from severely injured trauma patients. Patients were grouped to no P group without pneumonia or P group with pneumonia. Samples were obtained at admission to emergency department (ED) and one (Sadeghi-Bazargani et al. 2018) day prior to pneumonia. Samples from the equal post-injury days in the corresponding no P group were used. **b** Phagocytosis in CD16^+^ granulocytes after incubation with CC16 and neutralization of CXCR1 (CXCR1-ab), CXCR2 (CXCR2-ab) as well the corresponding isotype control antibodies (ctrl-ab), respectively. **c** Phagocytosis of immature CD16^dim^CD62L^bright^, (**d**) mature CD16^bright^CD62L^bright^ and (**e**) immunesuppressive CD16^bright^CD62L^dim^ granulocytes. Data are represented as mean ± SEM

The percentage of phagocytizing cells did not significantly change neither after the incubation of PMNL with IL-8 nor with sera from TP (Fig. 4 a).

With regard to the intensity of phagocytosis, there were neither significant changes in CC16 stimulated granulocytes nor upon CXCR1 or after CXCR2 neutralization (Fig. 4 b). Further evaluation of neutrophil subsets with regard to phagocytosis has shown that there were no significant changes in immature CD16^dim^CD62L^bright^, immune suppressive CD16^bright^CD62L^dim^ and CD16^bright^CD62L^bright^ neutrophils vs. ctrl (Fig. 4 d).

### Blockade of chemokine receptors do not change the CC16-induced reduction of apoptosis

**Fig. 5:**
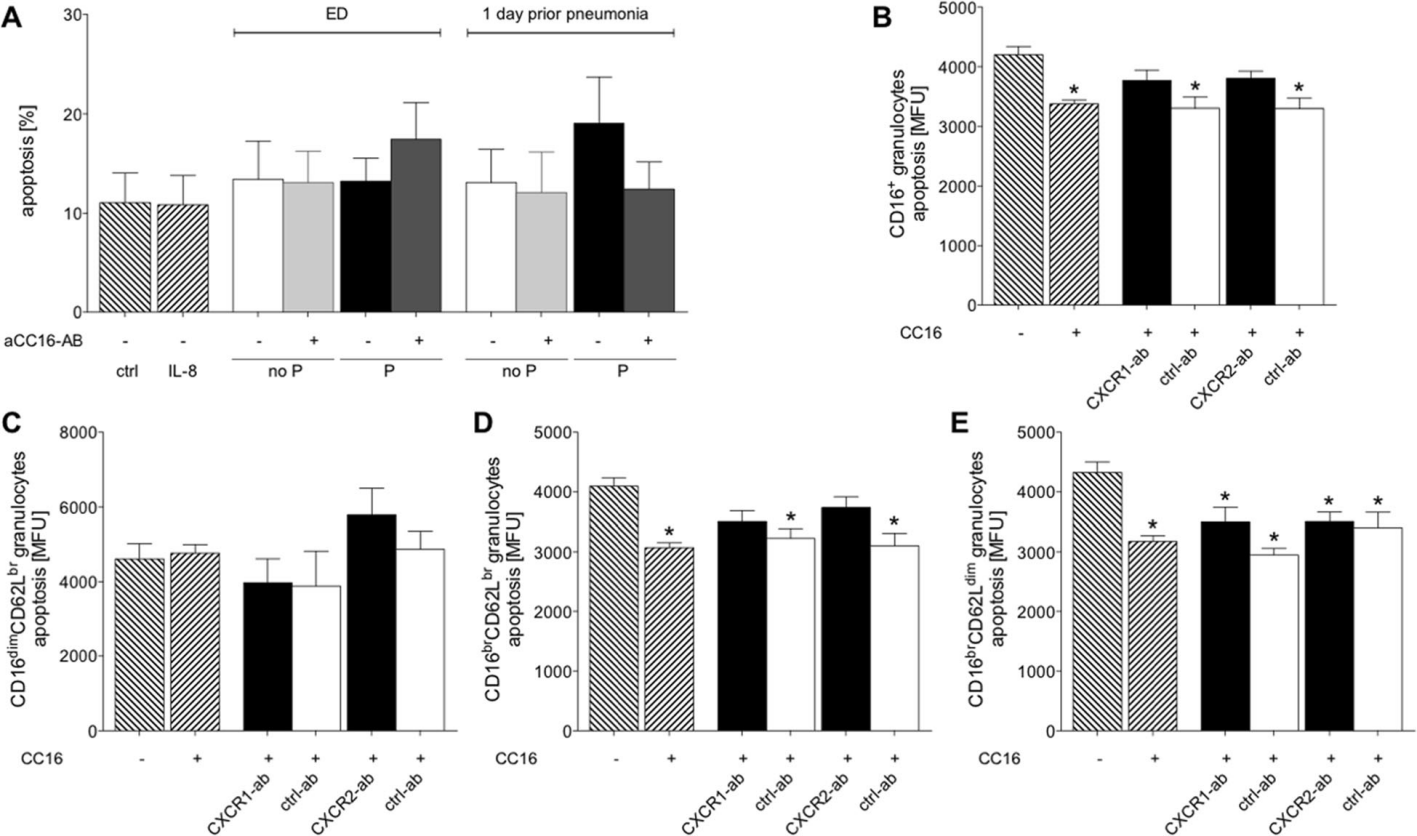
CC16-reduced apoptosis is not markedly changed by the blockade of chemokine receptors. **a** Apoptosis of PMNL after their incubation with IL-8 or sera samples from severely injured trauma patients. Patients were grouped to no P group without pneumonia or P group with pneumonia. Samples were obtained at admission to emergency department (ED) and one (Sadeghi-Bazargani et al. 2018) day prior to pneumonia. Samples from the equal post-injury days in the corresponding no P group were used. **b** Apoptosis in CD16^+^ granulocytes after incubation with CC16 and neutralization of CXCR1 (CXCR1-ab), CXCR2 (CXCR2-ab) as well the corresponding isotype control antibodies (ctrl-ab), respectively. **c** Apoptosis of immature CD16^dim^CD62L^bright^, (**d**) mature CD16^bright^CD62L^bright^ and (**e**) immunesuppressive CD16^bright^CD62L^dim^ granulocytes. Data are represented as mean ± SEM. *p* < 0.05: *: vs. ctrl

Incubation of isolated PMNL with IL-8 or with sera from TP did not markedly change the percentage of the apoptosis vs. untreated controls (Fig. 5 a). Neutralization of CC16 did not show any significant differences among the groups.

Treatment of isolated granulocytes with CC16 significantly reduced apoptosis vs. untreated ctrl of CD16^+^ granulocytes (*p* < 0.05, Fig. 5 b). Treatment of isolated granulocytes with neutralizing CXCR1 or CXCR2 antibodies diminished the CC16 induced reduction in apoptosis of CD16^+^ granulocytes (Fig. 5 b). Treatment of isolated granulocytes with corresponding isotype control antibodies resulted in significantly reduced apoptosis after incubation with CC16 vs. ctrl (*p* < 0.05, Fig. 5 b). Further evaluation of neutrophil subsets with regard to apoptosis has shown no significant changes among immature CD16^dim^CD62L^bright^ neutrophils. However, treatment with CC16 significantly reduced apoptosis in mature CD16^bright^CD62L^bright^ neutrophils, which has been abolished in CXCR1 as well as in CXCR2 neutralized samples (Fig. 5 d). With regard to immune suppressive CD16^bright^CD62L^dim^ neutrophils, a significantly reduced apoptosis in CC16 treated cells vs. ctrl has been detected (*p* < 0.05, Fig. 5 e). In this subset, CXCR1 and CXCR2 neutralization or incubation with isotype control antibodies did not markedly change the CC16 induced reduction of apoptosis (Fig. 5 e).

## Discussion

CC16 is increased in patients with pulmonary complications. Exposing isolated PMNL from HV to sera from patients with pneumonia increased migratory rates to IL-8 compared with patients without pneumonia. Furthermore, CC16-dependent decrease in IL-8 induced PMNL-migration emphasizes its role in neutrophil recruitment to the inflammatory site. This is in line with published data showing that CC16 reduced neutrophil migration upon stimulation with phospholipase A2 (Schrama et al. 2010). Similarly, CC16 inhibited IL-8 induced migration of equine neutrophils in a dose-dependent manner (Cote et al. 2014), although we did not observe any dose-dependency. However, since we applied much lower dosages of CC16, possibly that the narrow range of CC16 concentrations may be the reason for the lack of dose-dependent differences. The underlying mechanism of CC16-dependent decrease of PMNL migration has been studied in great extent. CC16 has a high potency to inhibit the neutrophil migration toward N-formyl-methionyl-leucyl-phenylalanin (fMLP) (Johansson et al. 2009). Therefore, CC16 may modulate the PMNL migration by antagonizing fMLP receptors. Considering our results, CC16 in sera from traumatized patients may modulate neutrophil migration via CD62L and potentially via other still unknown receptors.

Along with elevated PMNL migration following treatment with TP sera and consistent with our previous findings (Relja et al. 2016), the expression of adhesion molecules CD11b/CD18 was increased, whereas the CD62L surface presentation was decreased. An inappropriate expression of adhesion molecules on PMNL and their migration may be affected by pro- and anti-inflammatory mediators (Giannoudis et al. 2000). In traumatized patients with pneumonia, PMNL can downregulate the immune response by cleaving associated or essential receptors on the surface of both adaptive and innate immune cells (van den Berg et al. 2014). Less invasive transmigration of those cells has been observed following disruption of CD62L/CaM interaction by phosphorylation of CD62L at Ser 364 (Rzeniewicz et al. 2015). Apparent modulations of CD62L shedding on PMNL were related to the development of posttraumatic acute lung injury (Rainer et al. 2000). These findings are in line with the data presented in this manuscript. Weaver et al. have described that PMNL migration into injured cortex was notably increased at 24 h after trauma, and that this emigration could be neutralized by anti-CD11b antibody (Weaver et al. 2000). Increased expression of CD11b/CD18 on the PMNL surface may promote the binding to activated endothelia and, thus, enhanced penetration potency as well as emigration into surrounding tissues after trauma. Chishti et al. have shown reduced chemotaxis toward IL-8 in neutrophils from septic patients compared with healthy controls, whereby the surface expression of the chemokine receptor CXCR2 and the beta-integrin CD11b, but not CXCR1 was decreased and correlated with disease severity (Chishti et al. 2004). Involvement of CXC receptors in CC16-modulated functionality of neutrophils is in line with our findings. Similarly, enhanced neutrophilic migration after major trauma was associated with changes in the expression of their surface receptors such as up-regulation of CXCR1, or down-regulation of CD11b and CD18 in patients with ARDS (Bhatia et al. 2005).

Changes in general oxidative burst are in line with previous reports showing that pulmonary complications were characterized by increased neutrophilic respiratory burst activity (Hazeldine et al. 2014; Rivkind et al. 1991). In our study, oxidative burst of CD16^+^ granulocytes did not change upon neutralizing CXCR1 or CXCR2 compared to CC16-induced response. In line with this data, neutrophil-like human promyelocytic cells did not show differences in ROS-production with or without transducing CXCR1 gene after incubation with IL-8 (Kikuchi-Ueda et al. 2012). However, the effects of CC16 on oxidative burst of granulocytes were evident when analyzing notably the mature subset of neutrophils. It has been reported that the inhibition of CXCR2 reduced the superoxide production and NADPH activity in an angiotensin II or deoxycorticosterone acetate salt induced mouse hypertensive models (Wang et al. 2016). Therefore, it appears reasonable that CXC receptors may be involved in CC16 modulated ROS production predominantly in mature neutrophils after trauma. Yet, it remains to be noticed that different protocol such as e.g. seahorse mito stress test would provide a second line of evidence, and this must be considered in future studies.

Regarding phagocytosis, although increased ROS production and impaired phagocytic capacity of leukocytes is well described in traumatic brain injury (Liao et al. 2013) and spinal cord injury (Kanyilmaz et al. 2013), no changes were observed. Infectious complications e.g. pneumonia or ARDS seem to be associated with diminished neutrophilic phagocytosis and bacterial killing efficiency, with markedly higher decrease in trauma patients with ARDS compared with those with pneumonia and control (Mascellino et al. 2001). Whereas we have exposed isolated neutrophils from HV to sera obtained from traumatized patients, others have used isolated neutrophils from traumatized patients, which could explain such data differences. Furthermore, we did not stratify the patients according to their injury pattern, and thus, patients with several types of injuries including abdominal, head, extremities as well as thoracic traumata were included. Although it has been shown that phagocytosis of opsonized yeast by neutrophils reduced the expression of CXCR1 and CXCR2 (Doroshenko et al. 2002), we could not provide evidence for CXCR1 or CXCR2 involvement in phagocytosis in this experimental setting. A time-dependency of the observed effects as well as certain types of traumatic injuries to elaborate neutrophilic phagocytosis should be considered in larger studies.

CC16 protects lungs from cigarette smoke-induced injury by reducing NF-kappaB activation and alveolar cell apoptosis (Laucho-Contreras et al. 2015). Thus, CC16 can potentially prolong or shorten the longevity of neutrophils. However, it remains unclear, why there were no differences in apoptosis, since it is evident that inflammatory signals released after trauma do modulate apoptosis. The small sample size may explain why no differences were observed. It was shown that IL-1β, TNF-α, IL-6 or IFN-γ or bacterial products can interfere the physiologic process of apoptosis, and prolong the survival of PMNL after trauma (Colotta et al. 1992; Nolan et al. 2000). Moreover, in mechanically ventilated patients with ARDS, accelerated neutrophil apoptosis facilitated the resolution of inflammation in the preclinical models of lung inflammation. Taking together, the abnormal functions of neutrophils during severe trauma may cause a state of neutrophil paralysis (Phillipson and Kubes 2011; Alves-Filho et al. 2010) resulting in injury of host tissues (Phillipson and Kubes 2011). With regard to a potential mechanism, through upregulating the p53-CXCR2 axis, CXCR2 could reinforce the cellular senescence of cancer cells caused by resveratrol-induced replication and oxidative stress, and this inhibited apoptosis (Li et al. 2017). Our data show that CC16 modulated apoptosis of CD16^+^ granulocytes after neutralization of CXCR1 or CXCR2. Interestingly, the specific subsets of mature and hypersegmented, immunosuppressive cells after severe traumatic injury could reveal the influence of CC16 on their apoptosis rates via CXCR.

The expression of surface markers such as e.g. CXCR4 or CD62L can distinguish between the different neutrophil subsets (Pillay et al. 2012; Martin et al. 2003). As neutrophils age, they up-regulate cell surface expression of CXCR4 and acquire the ability to migrate towards CXCL12 (Nagase et al. 2002; Martin et al. 2003). Interestingly although apoptotic neutrophils still have a high expression of CXCR4, they cannot migrate to CXCL12 (Whyte et al. 1993). Thus, it can be differed between pre-apoptotic or “senescent” neutrophils and functionally and phenotypically distinct from apoptotic neutrophils, both expressing CXCR4 (Rankin 2010). Concomitant with CXCR4 up-regulation on ageing neutrophils, there is a decrease of the cell surface CXCR2 receptor expression, which itself plays an important role play in the recruitment of neutrophils to sites of inflammation, suggesting that senescent neutrophils have a reduced capacity for migration to these sites (Martin et al. 2003; Rankin 2010). With regard to the role of those cells in diseases, it was shown that neutrophils present in the synovial fluid of patients with inflammatory joint diseases and in the bronchoalveolar fluid of patients with chronic airways disease expressed high levels of CXCR4 (Bruhl et al. 2001; Hartl et al. 2008). Possibly, these cells age at this site of inflammation and do not re-enter the circulation for clearance. Here, we provide evidence for different responsiveness of the various neutrophil subsets to CC16 stimulation although for the phenotyping CD62L and not CXCR4 was applied. Tak et al. found that CD62L^dim^ human neutrophils could be also seen as a separate aged subpopulation during inflammation (Tak and Firestein 2001). They show that banded neutrophils appeared much earlier in blood than CD62L^dim^ and segmented neutrophils (Tak et al. 2017). In healthy volunteers administrated with endotoxin or severe trauma patients, a subpopulation of CD16^high^CD62L^low^ aged immunosuppressive neutrophils can be separated from whole human blood which was characterize by hypersegmented nuclei, a sign of aging change (Silvestre-Roig et al. 2019; Pillay et al. 2012). We also report that anti-inflammatory CC16 can induce functional alterations of this CD16^high^CD62L^low^ phenotype. It remains to be further studied in traumatic setting, if CD62L^dim^ cells are a truly separate subset that is recruited to the bloodstream in response to acute inflammation as suggested before (Tak et al. 2017). The depletion of the microbiota reduced the number of circulating aged neutrophils and dramatically improved the pathogenesis and inflammation-related organ damage in models of sickle cell disease or endotoxin-induced septic shock (Zhang et al. 2015). Thus, although neutrophils have been generally considered as a relatively homogeneous population for several decades, evidence for their true heterogeneity is emerging. This is also supported by differential impact of CC16 on the three subsets. As causative factors for their functional alterations their natural process of ageing and the replenishment by newly released neutrophils from the bone marrow, but also several other factors including e.g. genetics or microbiome are discussed.

Since in previous clinical study by Wutzler et al. it was shown that CC16 was a biomarker for detecting secondary respiratory complications in patients with multiple injuries (Wutzler et al. 2012), here, CC16 was not measured in the samples. Thus, this remains a limitation of the study, and it must be considered that CC16 values were not determined in the used samples. As a further limitation of the study, it remains to be considered that smoking, which is well-known to reduce CC16 levels was not documented. With this regard, several clinical studies have demonstrated that lower serum concentration of CC16 has been associated with the presence, risk, and progression of common obstructive lung diseases like asthma, COPD and smoking-related decline in lung function (Lam et al. 2018; Stenberg et al. 2018). The long-term Tucson Epidemiological Study of Airway Obstructive Diseases (TESAOD) further indicated that low serum concentration of CC16 was strongly associated with the mortality caused by cigarette smoke-related cancers, particularly lung cancer (Guerra et al. 2013). CC16 deficiency increases smoke-induced lung pathologies by its effects on epithelial cells, leukocytes, and fibroblasts in vivo (Laucho-Contreras et al. 2015). Interestingly, an experimental augmentation of CC16 levels in epithelial cells or smoke-exposed murine airways reduces inflammation and cellular injury (Pang et al. 2015; Pang et al. 2018). Thus, CC16 may play an important protective role in cigarette smoke-related diseases. Since we were unable to provide any information on the smoking behavior of included patients, we this remains a critical limitation of the present work and should be considered in future analyses.

## Conclusions

In general, these results confirm previous studies regarding the anti-inflammatory potential of CC16. However, several new issues about the effects of CC16 on PMNL such as potentially CXCR1- or CXCR2-mediated oxidative burst and/or apoptosis by CC16 in certain neutrophil subsets have been provided. Summarized, the apparently CC16 migration-reducing and oxidative stress-inducing effects on PMNL may be associated with the development of post-traumatic pneumonia. On the one hand, an impaired ability of neutrophils to eliminate pathogens but also pathogen-associated molecular patterns, and on the other hand increased injury to the local tissues enhancing the susceptibility to infections e.g. in the lungs are possible. However, these anti-inflammatory effects may also play a protective role under inflammatory conditions, preventing an exaggerated local immune response and exceeding infiltration of e.g. lungs with PMN. This remains to be elucidated in further studies.

## Data Availability

A truncated data set used and/or analysed during the current study is available from the corresponding author on reasonable request.

## Abbreviations

APC: Allophycocyanin
ARDS: Acute respiratory distress syndrome
CC16: Club Cell protein 16
CD: Cluster of differentiation
ctrl: control
CXCR: CXC chemokine receptors
ED: Emergency department
FACS: Fluorescence activated cell sorting
FITC: Fluorescein isothiocyanate
fMLP: N-formyl-methionyl-leucyl-phenylalanin
HV: Healthy volunteers
IL: Interleukin
ISS: Injury severity score
MFU: Mean fluorescence units
MOF: Multiple organ failure
P: Pneumonia
PE: Phycoerythrin
PMNL: Polymorphonuclear leukocytes
ROS: Reactive oxygen species
SEM: Standard error of the mean
TNF: Tumor necrosis factor
TP: Trauma patients
